# Combination of tumor antigen drainage and immune activation to promote a cancer-immunity cycle against glioblastoma

**DOI:** 10.1007/s00018-024-05300-5

**Published:** 2024-06-22

**Authors:** Han Xu, Xiaomei Zhao, Jincai Luo

**Affiliations:** Laboratory of Vascular Biology, Institute of Molecular Medicine, College of Future Technology, Beijing Key Laboratory of Cardiometabolic Molecular Medicine, Peking University, Beijing, 100871 China

**Keywords:** Meningeal lymphatic vessel, Glioblastoma, Antigen drainage, Immune activation, Cancer-immunity cycle, Tertiary lymphoid structure

## Abstract

While conventional cancer modalities, such as chemotherapy and radiotherapy, act through direct killing of tumor cells, cancer immunotherapy elicits potent anti-tumor immune responses thereby eliminating tumors. Nevertheless, promising outcomes have not been reported in patients with glioblastoma (GBM) likely due to the immune privileged status of the central nervous system and immunosuppressive micro-environment within GBM. In the past years, several exciting findings, such as the re-discovery of meningeal lymphatic vessels (MLVs), three-dimensional anatomical reconstruction of MLV networks, and the demonstration of the promotion of GBM immunosurveillance by lymphatic drainage enhancement, have revealed an intricate communication between the nervous and immune systems, and brought hope for the development of new GBM treatment. Based on conceptual framework of the updated cancer-immunity (CI) cycle, here we focus on GBM antigen drainage and immune activation, the early events in driving the CI cycle. We also discuss the implications of these findings for developing new therapeutic approaches in tackling fatal GBM in the future.

## Introduction

In adults, glioblastoma (GBM), the most common primary brain tumor, remains uniformly lethal with most surviving less than one year and merely 5% surviving beyond 5 years [1], while in childhood, GBM is the most common solid tumor and the leading cause of cancer-related death in this population, which averages 74% 5-year survival, but through the full age averages just 34%. [2] Currently, few therapeutic options exist for GBM outside of surgical resection, radiation therapy and chemotherapy to which GBM is often resistant. Immunotherapy, which is represented by immune checkpoint inhibition and adoptive T cell transfer, represents a conceptual revolution in the management of multiple cancer types [3–5], but unfortunately, it has failed to improve clinical outcomes in the patients with GBM. For example, in a recent randomized phase III clinical trial focusing on programmed cell death pathway inhibition in GBM, the anti-PD-1 therapy failed to prolong overall survival in these patients [6]. Similarly, initial tumor regression noted in a clinical trial adoptive T cell transfer for GBM was subsequently followed by disease progression [7]. Despite these negative results, several studies showed that some patient subsets exhibit prolonged survival following this form of ICB [8, 9]. Notably, in some case reports, neoadjuvant PD-1 blockade was shown to induce clinical benefit and elicit immunological responses in patients with recurrent GBM as compared with adjuvant immunotherapy [9]. Recent investigations indicated that chimeric antigen receptor T cell (CAR-T) therapy [10] and dendritic cells (DCs) vaccine [11] extended survival among some patients with GBM. To further improve the durability and effectiveness of antitumor immune response, it is necessary to systematically understand the intrinsic features of anti-GBM immunity and tumor microenvironment (TME). In this review, we summarize the current knowledge about GBM-specific antigen drainage, immune trafficking, and immune activation using the framework of the updated cancer-immunity (CI) cycle, and discuss potential strategies to enhance immunotherapy efficacy for GBM.

### A unique CI cycle in GBM

**Fig. 1:**
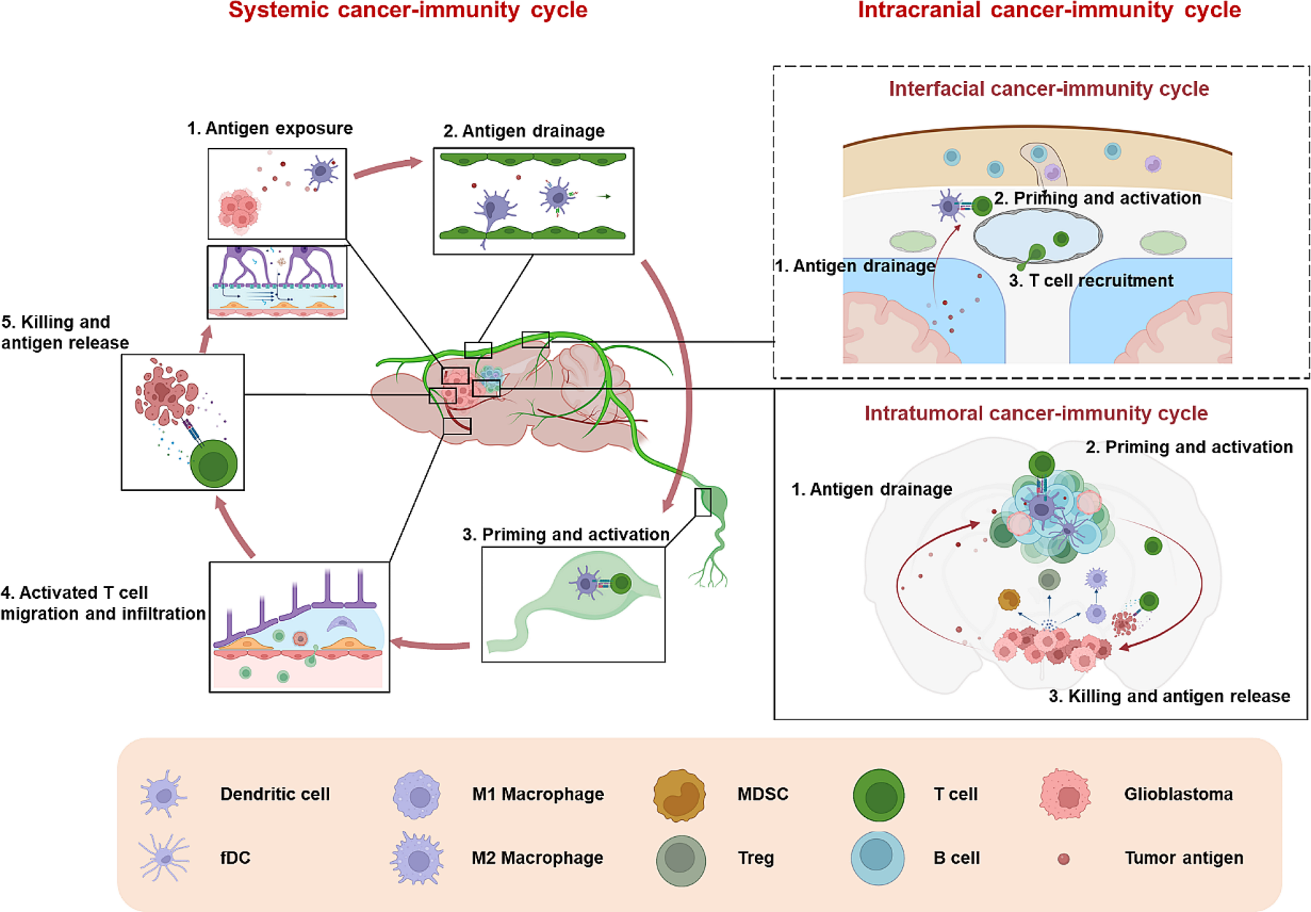
The non-canonical systemic and intracranial CI cycles in GBM. Activation of the anti-tumor immune response within LNs follows a distinct pathway (systemic CI cycle): antigen-presenting cells (APCs) loaded with antigens migrate into dCLNs *via* MLVs, where they present processed peptides to CD8^+^ T cells. Subsequently, activated CD8^+^ T cells migrate to tumor sites, where they execute their cytotoxic function by killing tumor cells [13, 40, 41]. Activation of the anti-tumor immune response within TLSs involves a series of coordinated events (intratumoral CI cycle): Tumor cells release tumor-specific and self-antigens. APCs within TLSs capture these antigens and present them to CD8^+^ T cells, thereby initiating the formation of effector anti-tumor cytotoxic T cells [36]. Activation of the anti-tumor immune response in meninges (interfacial CI cycle) has not been reported. However, considering that antigen presentation occurs at this CNS immune interface during neuroinflammation [39], we speculate meninges may also harbor the CI cycle

The CI cycle is comprised of a series of stepwise functional events including the antigen release of tumor cells, antigen presentation by DCs, priming and activation of T cells, trafficking of effector T cells to tumors, and infiltration of the T cells into tumors for recognizing and ultimately killing tumor cells [12, 13]. Very recently, the CI cycle theory has been updated by including a key role for the tumor microenvironment (TME), particularly DCs, in regulating and sustaining the anti-tumor T cell response [12, 13]. In GBM, the CI cycle is non-canonical as it includes two types: systemic and intracranial cycles, which interact on the meningeal interface and will be detailed below (Fig. 1).

Antigen exposure and drainage represent the initial stages of the systemic cycle. The absence of conventional lymphatic system in the brain parenchyma make the antigen drainage of GBM distinct from that in periphery [14, 15]. The glymphatic system enables the flow of cerebrospinal fluid (CSF) through perivascular spaces into the brain parenchyma, thereby facilitating exchange with interstitial fluid (ISF) and enabling an outflow of CNS-derived fluids and CSF/ISF waste solutes [16–18]. Since GBM-derived antigen was detectable CSF [19, 20], the glymphatic system may contribute to its enrichment in CSF and subsequent drainage to periphery. Interestingly, a recent study observed a reduced glymphatic clearance in GBM rats and further drain to the extracranial lymphatic vessels, speculating that reduced CSF drainage may contribute to reduced anti-tumoral T-cell activation and a weaker immunological response in GBM [20]. Notably, although DCs are negligible in healthy brain parenchyma [21], almost all subsets of DCs can be observed in the core lesions of gliomas [22]. Thus, released tumor antigens can also captured by DCs infiltrated in GBM [23]. The CSF containing antigens subsequently drains out of the skull directly into dural sinuses or into cervical lymph nodes (CLNs) along olfactory nerves penetrating the cribriform plate [24], whereas the trafficking routes of DCs remain unclear. Later on, the re-discovery of functional meningeal lymphatic vessels (MLVs), which extend into the meningeal tissue that wraps the entire CNS of mice [14, 15], fishes [25], primates [26], and humans [27], updated the routes of CSF antigen drainage and DC trafficking. MLVs have been shown to sample and drain CSF contents, including macromolecules, T-cells, and MHC II-expressing APCs, directly into the deep cervical lymph nodes (dCLNs) [14, 15, 28]. Besides, a recent study elegantly discovered an extended anterior MLV network around the cavernous sinus, with exit routes through the foramina of emissary veins [27]. Another study revealed a distinctive lymphatic plexus in the nasopharynx (NPLP) serving as a hub for CSF outflow through lymphatics from the cribriform plate and select other intracranial regions to dCLNs [29]. These present findings reveal an optimal pathway, namely the ISF-CSF-MLV-CLN pathway, facilitating communication from the CNS to the periphery. Notably, in GBM, current evidence suggests that soluble antigens and antigens loaded by APCs are primarily drained by MLVs in the dura mater from tumor sites to dCLNs [23, 30–32]. 

T cell priming and activation represent the central step of the CI cycle. DCs carry tumor antigens from intracranial tumors to dCLNs, where they prime naïve CD8^+^T cell [23]. Furthermore, resident DCs in CLNs can also capture soluble antigens drained from tumors [23]. Enhancing the trafficking of soluble antigens and DCs to dCLNs can elicit more robust T cell activation, thereby facilitating tumor immune responses [30–32]. The following step of CI cycle involves addressing the trafficking of immune cells into the CNS and their passage through the blood-brain barrier (BBB) [33]. Recruitment of activated T cells to the CNS involves a sequence of steps beginning with the adhesion of T cells with vascular endothelial cells [34]. Then, T cell roll along endothelial vessels and ultimately extravasate the endothelium, following a gradient of chemoattractant cytokines. After migrating through the BBB and entering the perivascular space, T cells must traverse the glia limitans [35]. The matrix metalloproteases secreted by T cells contribute to disrupt this layer, facilitating their entry into the brain parenchyma [35]. Here, they finally encounter tumor cells and mount an immune response. In summary, the distinct steps of T cell priming, activation, and migration within the CI cycle underscore its unique process and essential role against GBM.

In addition, as depicted in the right panel of Fig. 1, the intracranial immune cycle has been partially elucidated. Antigen release remains the initial phase of this immune cycle. However, unlike systemic CI cycle, tumor antigens or DCs do not require complex drainage pathways to access the periphery. In intratumoral CI cycle, T cells have the opportunity to interact with APCs (particularly DCs) located at the tumor sites, especially in tumor-associated tertiary lymphoid structures (TLSs) [36]. Following priming by DCs, T cells undergo expansion and differentiation, ultimately leading to direct cytotoxicity against tumor cells [13]. Furthermore, CSF interacts with the interface of the CNS through specialized channels, which may lead to the formation of additional CI cycles. For instance, CSF can ingress directly into skull bone marrow *via* dura-skull channels, establishing an interface with immune cells in this area [37]. Besides, arachnoid cuff exit (ACE) points, which represent the discontinuities in the arachnoid barrier around bridging veins, enable direct CSF and cellular exchange between the dura mater and the subarachnoid space [38]. These anatomical features enable the CNS borders to sense changes of antigens within the CSF, thereby facilitating immune surveillance and antigen presentation during instance of CNS inflammation. Notably, CNS-derived antigens in the cerebrospinal fluid accumulate around the dural sinuses, where they are captured by local APCs and subsequently presented to patrolling T cells. T cell recognition of CSF-derived antigens at this site promotes the development of tissue resident memory T cells and effector functions within the dural meninges [39]. Hence, the dural meninges may potentially serve as a site of immune activation in CI cycle. However, further research is warranted to substantiate this hypothesis.

**Fig. 2:**
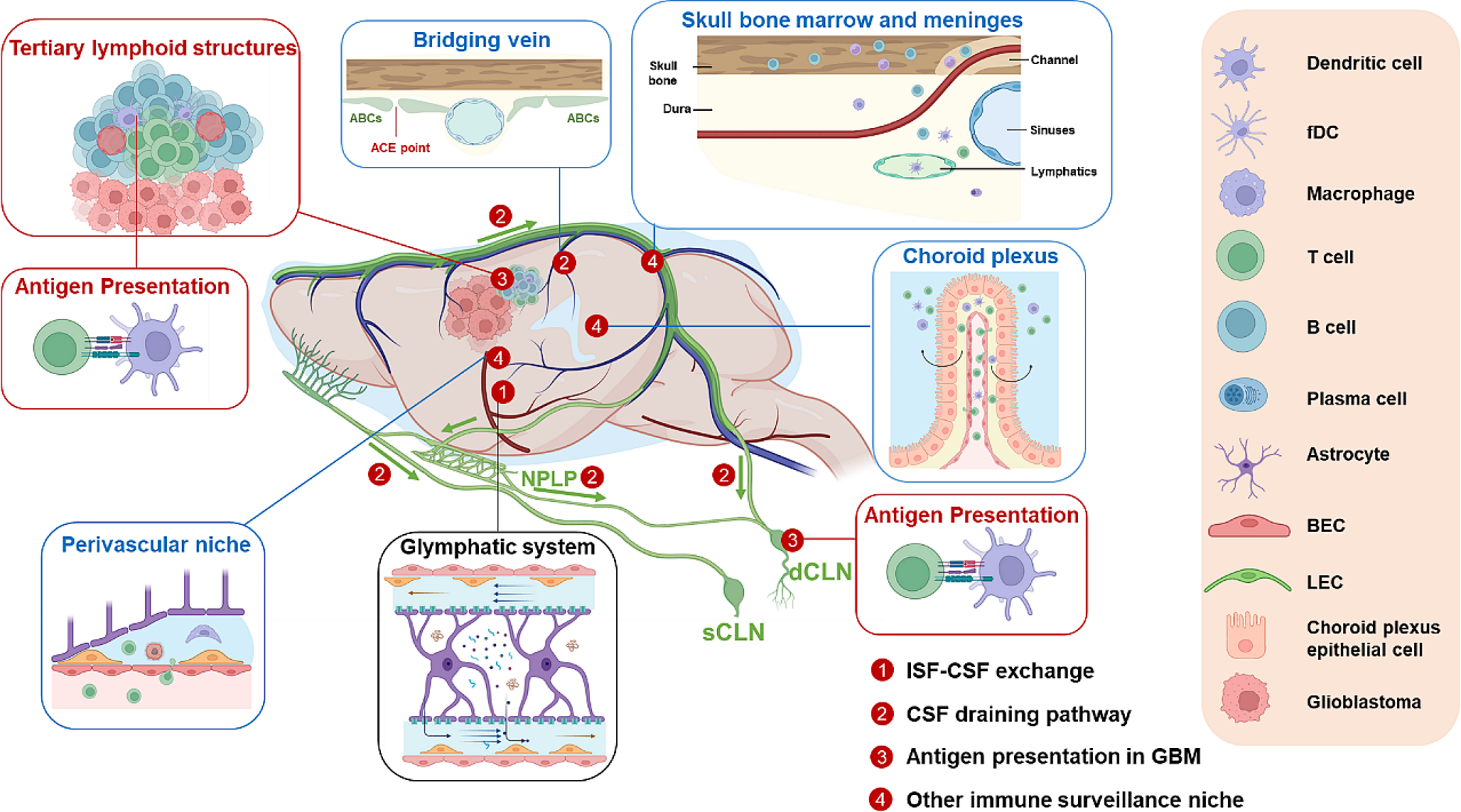
Pivotal sites for antigen drainage and immune activation in mice bearing glioblastoma. 1.The glymphatic system facilitates the movement of CSF through the brain parenchyma and its exchange with ISF [16, 18]. 2. Dorsal [137] and basal [12, 13] MLVs directly promote CSF drainage primarily in dCLNs. The NPLP serves as a central hub for CSF drainage to dCLNs [29], meanwhile cribriform plate lymphatics also contribute to CSF drainage in sCLNs [24]. 3. In GBM, the current evidence suggests that antigen presentation and activation primarily occur in dCLNs [24] and TLSs [25] located around the tumors. 4. The anatomical features in CNS borders, including the dural mater [38, 54, 138], skull [37], choroid plexus [139], and perivascular spaces [140] are recognized as specialized niches that facilitate immune surveillance and antigen presentation in some cases of CNS inflammation

Above all, based on the remarkable progress in the past few years, we illustrated a unique CI cycle in GBM. Notably, the activation step in the GBM CI cycle may occur not only in the dCLNs but also in tumor-associated lymphoid structures and potential interfaces within the CNS [13, 40, 41]. In the following sections, we will discuss individual events in the early steps of the CI cycle (Fig. 2), highlighting their complex nature: such as complex pathways of tumor antigen release and drainage, unclear trafficking routes of antigen-loaded DCs, multiple sites for antigen presentation to T cells, and tight barrier for effector T cells infiltration into tumor tissue.

### GBM-derived antigen drainage

Cancer rejection antigens, such as tumor-associated antigens (TAAs) and tumor-specific antigens (TSAs), are the targets of anti-tumor T cells [42]. Considering the unique immune microenvironment and poor response to immunotherapy, the nature and drainage pathway of these antigens of GBM have generated intense interest. TAAs are self-antigens encoded in the germline genome that are preferentially expressed in tumors, which are generally weakly immunogenic [42]. TSAs resulting from genetic aberration are highly immunogenic but are at low generation level in GBM due to the unique low tumor mutation burden in GBM [42–44]. Moreover, given the lack of lymphatic vessels in brain parenchyma, it is proposed that GBM antigens could first be enriched in CSF by ISF-CSF exchange in glymphatic system or loaded by APCs for subsequent draining by MLVs. In this section, recent progress in GBM soluble antigen drainage and antigen-loaded DCs trafficking is discussed.

### Soluble antigens drainage

The presence of GBM-derived soluble antigens in CSF appears to rely on the glymphatic system. CSF movement into the parenchyma drives convective interstitial fluid fluxes within the tissue. These fluxes flow toward the perivenous spaces surrounding the large deep veins [17]. The interstitial fluid with soluble GBM antigens is collected in the perivenous space from where it drains out of the brain toward the CLNs [16]. Though there is currently no direct evidence of brain TAAs exchange from the brain parenchyma into CSF by the glymphatic system, the clearance of injected soluble macromolecules or endogenous proteins from the parenchyma into the CSF by the glymphatic system has been confirmed [16, 45]. Additionally, clinical research has detected significantly higher levels of TAAs in the CSF of patients with primary or secondary brain tumors compared to control patients, suggesting that the glymphatic system contributes to the enrichment of tumor antigens in the CSF [19]. Studies in rodents bearing GBM demonstrated a reduced influx and delayed clearance of CSF tracer *via* the glymphatic system [20, 46]. Consistent with this, clinical data in glioma patients has shown impaired glymphatic function and decreased AQP4 expression in the astrocytes around the vessels in the tumor area [47, 48]. Although how brain tumors influence the glymphatic system and the impact on prognosis are not fully understood, reduced glymphatic flow may lead to fewer GBM antigens in CSF and subsequent reduced antigen draining by extracranial lymphatic vessels would likely cause less tumor-specific antigen exposure and a weakened anti-tumor immune response. Thus, treatment targeting the glymphatic system to restore glymphatic function in the cases of brain tumors has significant therapeutic potential in clinical practice. So far, several pharmacological modulations targeting functional glymphatic system components have proven to be effective [49] and may be employed in the future study of GBM antigen drainage.

Next, tumor antigens enriched in CSF drain into the CLNs for subsequent processing, presentation and leukocyte recognition. It has been demonstrated that ablation or augmentation of MLV function causes a significant alteration in brain TAAs draining into CLNs [30–32]. Hu and colleagues [31] used a fluorescein isothiocyanate (FITC)-dextran (> 70KDa) to mimic antigens exposed at the tumor site and demonstrated that dorsal meningeal lymphatics are the major functional path for draining tumor fluid. When they specifically photoablated the vessels with Visudyne and left both nasal and basal lymphatics intact, a significant reduction in FITC-dextran accumulation in the CLNs was found. Furthermore, diligent analysis of the anatomy of meningeal lymphatics has revealed that tumor cell injection into the brain induces both expansion of the dorsal meningeal lymphatics and strong lymphangiogenesis in dCLNs. These data together suggest that during GBM, MLVs undergo extensive remodeling to facilitate soluble TAAs draining into the dCLNs. This was further confirmed by experiments in which the ectopic expression of vascular endothelial growth factor C (VEGF-C) significantly improves the efficacy of immune checkpoint blockade (ICB) therapy [30, 31]. In mice injected with GL261-GFP^+^ or B16-GFP^+^ tumor cells into the cisterna magna, GFP^+^ tumor cells overlapped with MLVs and further invaded dCLNs, suggesting that MLVs serve as conduits for tumor cell draining to dCLNs [31]. Brain tumor cells can present peptide MHCI complex on the cell surface and be recognized as non-self by T cells, suggesting that these tumor cells in dCLNs may evoke an anti-tumor immune response. However, another research showed that cervical lymph nodes of mice with CT2A cells expressing blue fluorescence protein inoculated in striatum contained immune cells expressing brain tumor antigens (CD45^+^ BFP^+^ cells), while no tumor cells (CD45^−^ BFP^+^ cells) were detected in the node [30]. Several studies have also indicated that animals with GBM exhibit obstructed CSF outflow at the cribriform, providing further evidence supporting the notion that meningeal lymphatic vessels serve as the primary pathway for antigen clearance [20, 46]. However, we have to point out shortcomings in research on GBM antigen draining by MLVs. Although Hu et al. ingeniously designed experiments using fluorescent molecules injected exogenously to mimic antigens, direct evidence showing GBM antigens draining into dCLNs by MLVs is still lacking. Besides, though tumor cells injected in CSF can be detected in dCLNs, there is a lack of supporting evidence for metastasis of orthotopically injected tumor cells to the lymph node.

### Antigens-loaded DCs trafficking

As noted above, GBM antigens can also be delivered to certain immune hubs like CLNs by loading in APCs. Professional APCs include DCs, macrophages (microglia in the CNS), and B cells, characterized by their expression of major histocompatibility class II (MHC II) and their ability to process and present antigens to T cells. In the “systemic” CI cycle, where the priming and activation steps occur in LNs, DCs have been extensively studied for its capability in migration with antigens. Current studies observed DCs located at meninges, choroid plexus and perivascular spaces [50, 51] During GBM, these DCs may be attracted to the brain parenchyma to take up antigens and they subsequently migrate to tumor-draining dCLNs to present antigens [52]. In mice bearing GBM, a method has been developed to illustrate this process by intratumoral injection of 0.5 μm FITC-labeled beads that are too large to flow into lymphatic vessels and instead must be taken up by DCs around the tumor before being transported to dCLNs [31]. In dorsal MLV-defective mice, a dramatic reduction of CD11c^+^MHC II^+^FITC^+^ cells in the dCLNs has been reported, and consistent with this, the trafficking of DCs loading FITC-labeled beads to dCLNs is markedly greater in the group with VEGF-C induced MLVs extension than in Vector group [31]. This was further confirmed by Zhou and colleagues [32] who showed that the percentage of FITC^+^ DCs increased later in the CLNs of mice with VEGF-C-overexpressing gliomas after radiotherapy. In peripheral tissue, local primary lymphatic vessels play a critical role in DC trafficking by C-C motif chemokine ligand 21 (CCL21) secreted by capillary lymphatic endothelial cells (LECs). Besides, these capillary LECs are connected by “button”-like junctions to form a discontinuous layer to facilitate immune cell entry [53]. Similarly, MLVs at the dura mater share characteristics with primary lymphatics and also express CCL21, regulating DC trafficking to dCLNs on the CCL21/ C-C motif chemokin receptor (CCR7) axis [14, 15]. Furthermore, utilizing multiphoton microscopy to track fluorescent-labeled lymphatic vessel endothelial hyaluronan receptor-1 antibodies, Louveau et al. [54] demonstrated extensions of meningeal lymphatics along the transverse sinus and upper part of the superior sagittal sinus as ‘hot spots’ where tracers accumulated after i.c.m. injection. This suggests that the extensions of meningeal lymphatics serve as potential entry points for fluid macromolecules and immune cells from the CNS into peripheral primary lymphatics. Additionally, in the most recent research, discontinuities in the arachnoid barrier around bridging veins have been identified as direct connections between the dura mater and subarachnoid space. These connections permit the exchange of fluids and molecules between the subarachnoid space and the dura, as well as the entry of immune cells into the subarachnoid space [38]. Meningeal lymphatic endothelial cell-derived CCL21 is significantly increased along with enhanced meningeal lymphangiogeneis by VEGF-C, and the administration of anti-CCL21 or CCR7 antibodies leads to a failure in benefiting from VEGF-C [31]. These data together suggest an important role of dural lymphatics in modulating CCL21/CCR7-dependent trafficking of DCs containing GBM antigens. Also enhanced draining of antigens loaded by DC leads to better efficacy when VEGF-C is combined with other treatments, including cytotoxic T lymphocyte-associated protein 4 (CTLA-4), the PD-1 blockade and radiotherapy in mice bearing GBM [30–32]. These data demonstrate CSF-MLV-CLN as a primary route for both soluble antigens and antigens loaded by DCs to reach the periphery. Importantly, it highlights their integral contribution to the CI cycle, subsequently igniting the anti-GBM immune response.

In addition to MLVs, several pathways for CSF efflux have been demonstrated by previous studies. CSF efflux from subarachnoid space directly into dural venous sinuses through arachnoid projections and along outwardly-projecting cranial nerves, especially along the olfactory nerve, which is closely associated with cribriform plate lymphatics. The latest research has further elucidated the route of CSF drainage from the subarachnoid spaces to extracranial lymphatics. It reveals that CSF from the anterior and middle cranial regions of the subarachnoid space, including the cribriform plate, exits through the nasopharyngeal lymphatic plexus to reach the deep cervical lymph nodes (dCLNs) [29]. Though there is a lack of evidence about the relationship between glioma antigen drainage and cribriform plate lymphatics, recent achievements in neuroinflammation have demonstrated its potential as it expands in mice bearing experimental autoimmune encephalomyelitis (EAE) and recent single-cell-RNA sequencing have revealed the upregulation of genes involved in antigen presentation and cell adhesion [55, 56]. Notably, recent studies also reveal the dural sinuses and meninges as pioneer sites of contact with glioma soluble antigens and antigen-loaded DCs [39, 57]. With evidence showing that immune cell aggregation and the antigen presentation process take place in the dural sinuses and meninges, these two places have been identified as CNS immune niches and play important roles in neuroinflammation [50], and their role in regulation of anti-GBM immunity is a promising direction that remains to be explored.

### The activation of anti-GBM immunity

The CI cycle provides a theoretical framework to illustrate the process of the anti-tumor immune response. The anti-tumor immune activation processes commonly occur in the tumor microenvironment [58], tdLNs [59], and TLSs [60]. Recent updates to this model emphasize the importance of tumor’s immunological phenotype [13]. GBM exhibits reduced numbers of effective tumor-infiltrating CD8^+^ T lymphocytes and the presence of multiple immunosuppressive cell populations, such as tumor-associated macrophages (TAMs) [61, 62], regulatory T cells (Tregs) [63], and myeloid-derived suppressor cells (MDSCs) [64, 65] in the TME. Recent research revealed that naïve T cells are found sequestered in large numbers in the bone marrow in GBM mice and patients, resulting T cell deficiency in the blood and lymphoid organs [66]. These factors underscore the designation of GBM as a “cold tumor”, highlighting the pivotal role of immune activation in the efficacious management of GBM. Targeting specific immune cell populations to ameliorate the immunosuppressive microenvironment emerges as a critical strategy for enhancing therapeutic outcomes in GBM [58]. 

TAMs constitute 30-50% of the immune cells in GBM [67], comprising approximately 15% intrinsic microglia and 85% monocyte cells [62]. Traditionally, TAMs in glioma have been demonstrated to contribute to immune evasion and promote tumor proliferation [68], hindering the effectiveness of immune surveillance. Whereas, TAMs exhibit high plasticity, enabling them to polarize towards the pro-inflammatory subtype and increase its proportion [69]. This polarization helps remodel the immunosuppressive TME of gliomas, potentially enhancing the efficacy of glioma treatment. For instance, PD-1^+^ M2-like macrophages exhibit impaired phagocytic function, which can be reversed by PD-L1 blockade [70]. Knockdown of PD-L1 in GBM has been shown to upregulate M1-like populations and downregulate M2-like populations, thereby inhibiting tumor cell invasion and migration [71, 72]. The combination of IL-6 inhibition and CD40 stimulation effectively reversed macrophage-mediated tumor immunosuppression, enhanced T-cell activation, sensitized tumors to checkpoint blockade, and significantly prolonged animal survival in GBM models [73]. The Tregs population, despite its relatively low abundance within the glioma immune cells, exhibits potent immunosuppressive capabilities [74]. GBM patients presented significantly higher frequency of Tregs both in peripheral blood and tumor [75]. The increase in Tregs abundance correlates with a decrease in T cell cytotoxicity [76]. While targeting glucocorticoid-induced TNFR-related receptor (GITR) in Treg cells promoted CD4^+^ Tregs differentiation into CD4^+^ effector T cells, attenuated Treg cell-mediated suppression of anti-tumor immune response, and induced robust anti-tumor effector cells in GBM [63]. MDSCs have been demonstrated to inhibit T cell function *via* multiple mechanisms, infiltrating the glioma microenvironment and significantly contributing to tumor progression. In glioma patients, the intratumoral density of MDSCs increased during glioma progression and correlated with poor patient survival [77]. Study has demonstrated low dose 5-FU selectively depletes MDSCs, leading to prolonged survival in glioma mouse models [64]. Clinical research also indicated that the orally bioavailable 5-FU prodrug in combination with bevacizumab, reduces the circulating levels of MDSCs in GBM patients [78]. Additionally, systemic administration of anti-CCL2 antibodies can block recruitment and decrease the number of MDSCs in the TME, providing significant survival benefits in mouse glioma models [79].

Except for these immunosuppressive cells, DCs are also present in the TME. However, both the abundance and functionality of DCs are impaired in GBM, and their immunological relevance in tumor sites remains poorly understood [80]. A recent study elucidated the crucial role of conventional dendritic cells (cDC) in GBM, emphasizing their involvement in priming peripheral T cells, antigen presentation and T cell activation within the TME [81]. It also demonstrated that the presence of 2-hydroxy glutarate (2-HG) in IDH-mutant gliomas impairs the differentiation of monocytes into cDC, reducing their antigen-presenting ability and altering in the TME. The finding suggests that distinct subtypes of GBM and oncogenic metabolites may disrupt with the anti-tumor immune response mediated by DCs. Consist with this, another investigation illustrated that stem-like CD8^+^T are present in the unique APC niches and closely interacted with CD11c^+^ DCs within GBM tumors [36]. The APC niches has been shown to support the maintenance and differentiation of stem-like CD8^+^T cells in peripheral tumors, implying a potential similar role within GBM [82]. Notably, the recent concept of the CI cycle [13] has highlighted DCs as critical not only for initiating T cell responses early in the cycle but also for sustaining them. Therefore, the recognition of the importance of modulating DC activation or maturation in driving the CI cycle is increasing, potentially providing a promising therapeutic target.

In addition to the cells mentioned above, there are other immune cells present at the tumor site orchestrating to GBM immunity and we summarize these cells in Table 1. Despite the notable progress achieved, the immunosuppressive microenvironment within brain tumors continues to pose challenges for eliciting an immune response. Given the crucial role of immune activation in GBM, there’s a growing interest in exploring alternative avenues for immune activation such as tdLNs or TLSs adjacent to the tumor [36, 83]. In these immune activation sites, stromal cells, fibroblastic reticular cells, high endothelial venules, and lymphatic vessels provide a specialized niche to optimize immune cell-cell contacts such as B cells, T cells, and DCs [84]. This intricately regulated interplay between APCs and T cells [85] facilitates the generation of pathogen-specific immunologic effector pathways, the development of immunologic memory, and the maintenance of host immune homeostasis [59].

**Table 1:** Summary of pivotal factors targeting immune cells and stromal cells in GBM immunity based on existing literature and sequencing data. Immune cells promoting GBM progression: Tregs, MDSC, B cell and Macrophage/microglia. Immune cells inhibiting GBM growth: CD8 T cell, CD4 T cell (excluding Tregs), DC and Macrophage/microglia. Stromal cells: BEC, LEC and HEV

	Cell type	Molecule	Function	Ref
Immune cells	**CD8 T cell**	CD161	Inhibitory receptor	[86]
		IL-7	Increasing CD8 T cells	[87]
		CXCL14	Promoting CD8^+^ T cell recruitment	[88]
		IL-10	Leading to terminal exhaustion of T cells	[89]
		CD39	Promoting CD8 T cell dysfunction	[90]
	**Tregs**	IL-12	Causing a decrease in Foxp3 + Tregs	[91]
		CCL2	Recruiting Tregs	[92]
		GITR	Promoting Treg differentiation into CD4 effector T cell	[63]
	**CD4 T cell**	IL-12	Breaking T-cell inhibition by TGF-β2	[93]
	**DC**	CCL21	Inducing CCR7^+^ DCs drainage	[31, 32]
		Sarcosine	Inducing DC trafficking	[94]
		TLR3	Promoting the activation of DCs	[95]
	**MDSC**	CCL2	Recruiting MDSCs	[92]
	**B cell**	CD40	Inducing expansion of suppressive CD11b + B cells	[83]
	**Macrophage/microglia**	CSF-1/CSF-1R	Maintaining M2 TAMs in glioma microenvironment	[96]
		CCL2	Recruiting TAM	[97]
		AHR	Promoting CCR2 expression and driving TAM recruitment	[90]
		CD73	Producing adenosine to protecet tumor from immune surveillance	[98]
		CD40	Reversing macrophage-mediated tumor immunosuppression	[73]
		Galetin-9	Driving macrophage M2 polarization	[99]
		FGF20	Regulating macrophage function and exerting anti-inflammatory effects	[100]
		Osteopontin	Maintaining M2 macrophage phenotype.	[101]
Stromal cells	**BEC**	ELTD1	Impairing vessel function	[102]
		TGF-β	Downregulating CAM-expression and impeding T cell transmigration	[103]
	**LEC**	VEGF-C	Inducing lymphatic vessels expansion	[31]
	**HEV**	LTαβ	Promoting HEV formation	[36]
		LIGHT	Inducing TLSs and HEV formation	36]

### Cervical lymph nodes are pivotal in the GBM immune response

The tdLNs have been quite extensively investigated as a pivotal component of CI cycle in the periphery [104], while their roles in GBM have only recently been elucidated. In GBM, dCLNs exhibit an enrichment of T cells specific to tumor antigens, from both endogenous and exogenous sources [30], thereby indicating the activation of a robust tumor-specific immune response. DC trafficking from tumors to tdLNs has been shown to be important for antitumor immunity. Strategies aimed at enhancing these processes can augment the effects of ICB in mouse models [105]. Consistent with this, enhancing meningeal lymphatic drainage through the overexpression of VEGF-C can significantly promote the proportion of tumor-specific T cells, characterized by tetramer-positive CD8^+^T cell populations, in dCLNs and enhance ICB therapy efficency. VEGF-C treatment also induces changes in T cell phenotypes and functionality. This results in an increase of functional T cells that produce various cytokines, including tumor nerosis factor-α, interferon-γ, granzyme B, and interleukin-2, for killing tumors, and show more efficient immune activation in the dCLNs. It is noteworthy that Tregs have been demonstrated to impede the anti-tumor response in GBM, as evidenced by studies in both mouses models and patients [106]. VEGF-C treatment, while not impacting the percentage of Tregs, increased the ratio of CD8^+^Ki67^+^T cells to Tregs in both CLNs and tumor sites, indicating an enhancement of immune microenvironment [31]. Another crucial aspect of immune activation is the establishment of specific immune memory, which provides enduring protection against subsequent antigenic challenges after the acute immune response terminates. Assessment of the durability of the immune response against GBM in mice treated with VEGF-C revealed that mice rejecting an intracranial tumor rechallenge with GL261 in the flank exhibit no detectable tumors and demonstrate long-term systemic memory responses [30]. The efficacy of anti-tumor treatments, including ICB [30, 31] and radiotherapy [32], is significantly compromised following dCLN ligation or excision surgery. These data indicate that the dCLNs are crucial sites for antigen presentation and activation for GBM. Enhancing the functionality of the MLV-CLN system can elicit a robust and enduring T-cell-mediated immune response against GBM.

Recent studies have shown that the lymphatic vasculature is more than a passive conduit system. Tumor-associated LECs exhibit special features that control the egress of T cells from tumors [107]. Tumor-associated lymphatic vessels sequester CD8^+^ T cells at the tumor periphery, thereby increasing the probability of exit in a CXCL12–CXCR4-dependent manner in melanoma. The surface expression of CXCR4 on CD8^+^ T cells is modulated by antigen encounter, which consequently affects their susceptibility to CXCL12. Blocking CD8^+^ T cell egress through this pathway alone significantly improves local tumor control and enhances response to ICB. These findings suggest that the lymphatic vasculature plays a crucial role in shaping the diversity and functional state of the intratumoral CD8^+^ T cell repertoire, which highlights the potential of targeting CD8^+^ T cell egress as a control point for enhancing immunotherapy response in GBM. LN LECs also serve as antigen-presenting cells by expressing and presenting self- or non-self- antigens on MHC I and II molecules [108–110], or acquiring peptide-MHC II complexes from DCs [111, 112]. Though LECs as antigen-presenting cells are primarily associated with peripheral tolerance, some studies have unveiled their participation in CD8^+^ T cell priming and cancer progression [113–116]. This phenomenon is not limited to the periphery alone. In EAE model in the CNS, cribriform plate LECs have been demonstrated to enhance their capacity for binding CD11c^high^ CD11b DCs and CD4 T cells. They also internalize CNS-derived antigens, express MHC II, and upregulate immunoregulatory proteins such as PD-L1 in an interferon-γ-dependent manner. Furthermore, they can functionally present CNS-derived antigen to activate antigen-specific CD4 T cells [56]. These findings shed light on an immunoregulatory niche located near the cribriform plate within the lymphatics, which has been previously overlooked. Notably, the antigen-presenting related genes (MHC family) of LECs in MLVs are upregulated compared to those from the diaphragm and skin, indicating a unique immune function [54]. Consistent with this, several stages associated with the immune response, including the immune effector process, antigen processing and presentation, are significantly activated in LECs in MLVs in mice bearing GBM. The specific functions of these activated LECs require further investigation in near future [31]. These data indicate that the meningeal lymphatics are a potential immune activation niche, whereas more evidence is needed in GBM patients to corroborate these observations.

Although these findings were obtained using experimental tumor models with GBM cell lines, they shed a new light into the mechanism underlying GBM immunity and provide the MLV-CLNs pathways as a potential target for glioma treatment.

### Immune activation in TLSs

The updated cancer-immune cycle theory identifies the immune response within TLSs as a “subcycle” that plays a pivotal role in immune activation. However, its specific role in GBM remains largely unexplored. Recent studies showed that TLS formation is detectable in GBM both in humans and mice [36, 83]. In mouse GBM, TLSs exhibit a composition comprising B cells, T cells, DCs, fDCs, and blood endothelial cell (BEC), resembling those found in peripheral tumors [117], albeit lacking LECs. Notably, the promotion of TLS formation in GBM enhanced T cell priming, facilitated their infiltration into the tumor, and ultimately improved survival outcomes in mice [36]. This observation aligns with the favorable role of TLSs in the prognosis of peripheral tumors in clinical settings [84], including lung [118], colorectal [119], and pancreatic cancer [120]. Mechanistically, TLS formation in GBM was induced by lymphotoxin (LT)αβ or tumor necrosis factor superfamily member 14 (TNFSF14/LIGHT),^36^ suggesting the utilization of a similar mechanism to that in periphery. Interestingly, TLSs in mice are typically situated in proximity to the meninges, specifically around the cortex or adjacent to choroid plexuses, in close association with GBM tissue [36]. Similarly, TLSs, found in a subset of human WHO grade II-IV gliomas, are most frequently found in close proximity to meningeal tissue, but are also found in the white matter (close to the tumor bulk) or directly within the tumor tissue. Such a unique location of GBM TLSs could be due to the lack of LECs. The distribution of LECs in the CNS is highly specific, primarily localized to the meningeal lymphatic vessels and the recently discovered subarachnoid lymphatic-like membrane [121] in meninges. This specific distribution potentially elucidates the occurrence of TLSs lacking LECs in both GBM patients and mice, providing insight into their preferential localization within these anatomical sites.

Additionally, several recent studies have provided compelling evidence for the value of TLSs in predicting the response to immunotherapy. Therapeutic vaccination with an irradiated pancreatic tumor vaccine in conjunction with chemotherapy results in the formation of TLSs in a significant majority of patients with pancreatic cancers in the clinic [122]. The presence of TLSs in pretreatment biopsies of melanoma, renal cell carcinoma, soft tissue sarcoma, and urothelial carcinoma demonstrated a significant correlation with favorable outcomes following PD-1 [123, 124 or PD-1/CTLA-4 blockade [125]. Specifically, the combination of PD-L1 blockade with antiangiogenic therapies (LIGHT) results in TLS formation, increased CD8 T cell stimulation, and ultimately tumor destruction in mice [126]. Consistent with this, LIGHT treatment induces tumor-associated HEVs and T cell-rich TLSs, thereby improving the therapeutic effect of PD-1 checkpoint blockade in αPD-1-resistant murine glioma [36]. Moreover, It is worth noting that certain cell types within TLSs may contribute to tumor progression [60]. A recent study indicated that αCD40 stimulation of B cells promotes the formation of TLSs in mice bearing GBM by upregulating *Lta*, leading to the expansion of suppressive CD11b^+^ B cells and impairing T cell responses, which is consistent with an observation in a peripheral tumor [127]. Meanwhile, TLSs resident Tregs have been proven to induce tumor progression in periphery tumor as well [128]. In GBM, CD103^+^Tregs underlied resistance to radio-immunotherapy and impair CD8^+^T cell activation. Tregs targeting elicited TLS formation, enhances CD4^+^ and CD8^+^ T cell frequency and function and unleashes radio-immunotherapeutic efficacy [129]. Given the established correlation between TLSs and anti-tumor response, exploiting TLS induction and associated cell types emerges as an attractive therapeutic strategy in patients with GBM.

Overall, the findings of TLS formation in GBM emphases a potential “intracranial” CI cycle and hold promise for enhancing the poor therapeutic efficacy in GBM. Additionally, recent studies [130, 131] have shown that the meninges serve as niches for the development of immature B cells that migrate through microchannels from the skull bone marrow. This phenomenon likely arises from stromal cells containing developmental ligands, including CXCL12 and IL-7, around the dural sinuses. This exposes immature self-reactive B cells to CNS antigens, inducing immune tolerance and thus avoiding autoimmunity. Disruption of the local CXCL12-CXCR4 axis in the bone marrow also results in rapid recruitment of monocytes and neutrophils into the meninges in EAE models [37]. These studies underscore the pivotal role of these skull marrow-derived cells in the recognition of CNS perturbations. However, how do these skull marrow-derived cells shape antitumor immunity? Do skull marrow-derived cells constitute a distinct immune population in meninges within the tumor microenvironment, as in mouse models of CNS autoimmunity? Most recently, dural-associated lymphoid tissues (DALT) surrounding the rostral-rhinal confluence of the sinuses, which interface with the skull bone marrow, have been identified [132]. Immune aggregates were present in DALT during homeostasis and expanded with age or after challenge with systemic or nasal antigens. Notably, TLSs in meninges have also been described and believed to exacerbate immune responses against CNS antigens in CNS inflammation [133–135], chronic neurodegeneration, and spinal cord injury [136].This finding highlights the emergence of TLSs comprised of immune cells in the meninges during inflammatory conditions, potentially providing additional evidence for the crucial role of meningeal TLSs in CI cycle.

## Concluding remark

While the CI cycle theory exquisitely depicts the general process of the anticancer immune response against solid tumors, brain-specific aspects should be taken into consideration to understand the cycle’s individual steps and how they interconnect in GBM immunity. The CI cycle in GBM appears to be both intracranial and systemic, which is initiated by glymphatic convective bulk flow and MLV drainage. Notably, antigen drainage and DC maturation or trafficking, and subsequent T cell activations are emerging as key elements in driving the cycle. Conceivably, these new insights imply potential targets for therapeutic intervention.

Nevertheless, many important questions remain to be addressed. For example, whether the CNS borders are also important sites for immune activation in GBM like in neuroinflammatory disorders? What are the exact pathways by which GBM tissue-derived antigens and DCs exit the brain? Similarly, how does GBM manipulate the surveillance system to avoid the expression and detection of tumor antigens? Furthermore, both DCs and TLSs are located near tumor tissues. What are the differences in the types and functions of DCs in TLSs compared to those in tumors, and what are their regulatory mechanisms? More importantly, how and where are T cells activated by GBM antigens and get through the BBB to effectively kill tumor cells? Additionally, above studies focused on T cells in GBM. How about the role of anti-tumor B cells in the process? Given a substantial population of B cells in the skull bone marrow and meninges [130, 131], it is worthy investigating the role of B cells in GBM. These questions and many others will be addressed in the future using currently available and state-of-the art methodologies, such as those that are able to visualize and quantify immune cell populations and their spatial relationships at high resolution both in situ and at the organismal scale. The answers to the above questions may provide guidance on the development of novel immunotherapies for GBM.

## Data Availability

The manuscript has no associated data.
